# Social Presence: Conceptualization and Measurement

**DOI:** 10.1007/s10648-021-09623-8

**Published:** 2021-06-22

**Authors:** Karel Kreijns, Kate Xu, Joshua Weidlich

**Affiliations:** 1 Faculty of Educational Sciences, Open Universiteit, Valkenburgerweg 177, 6419 Heerlen, AT Netherlands; 2 Educational Technologies, DIPF | Leibniz Institute for Research and Information in Education, Rostocker Straße 6, 60323 Frankfurt am Main, Germany

**Keywords:** Social presence definition, Social presence measurement, Social space, Sociability

## Abstract

Social presence is an important construct in online group learning. It influences the way how social interaction unfolds online and affects learning and social outcomes. However, what precisely social presence is has been under debate, as presently a plethora of different definitions and measures exist preventing the development of a coherent research field regarding social presence and its defining role in online group learning. To solve the issue, we went back to the original social presence theory as devised by the communication researchers Short et al. (1976) to show that although they had a clear idea of social presence—namely “realness” of other persons in the interaction—their definition is ambiguous, not operationalizable, and the measurement of it questionable. We, therefore, disentangled their social presence theory and (1) reformulated the social presence definition to enable an operationalization in line with the previous conceptualization of social presence; (2) departed from the technological determinism of social presence; and (3) identified two other constructs closely linked to social presence, namely, sociability (as a medium attribute) and social space (as a group attribute). By reformulating the definition of social presence and by linking it to social space and sociability, we hope to contribute to a more coherent line of social presence research and to better understand interpersonal communication, group learning, and group dynamics when learning and working together in an online setting.

## Introduction

Quite a number of educational researchers investigating online distance education consider social presence an important construct in online group learning (OGL). Group learning refers to the “instructional use of small groups so that students work together to maximize their own and each other’s learning” (Johnson et al., 2014, p. 87). It is “an activity encouraging knowledge construction through mechanisms such as belief revision, conceptual change, externalizing knowledge and opinions, self-explanations, co-construction of knowledge and reflection” (Veerman, 2000, p. 68). In group learning, success is achieved only when other group members are also successful. Research on face-to-face group learning has shown numerous advantages of group learning over competitive (success is only achieved if others fail) or individual learning (success is achieved independent of others). A meta-study of 685 separate studies conducted by Johnson and Johnson (2014) confirmed that working together to achieve common group goals led to more higher-level reasoning, more frequent generation of new ideas and solutions (i.e., process gain), and transfer from group performance to individual performance (i.e., group-to-individual transfer). Hence, the growing interest in group learning and the application of it in online settings as well in order to create online group learning (Kreijns et al., 2021).

Social presence is associated with the use of computer-mediated communication (CMC) tools and electronic platforms for OGL in terms of the degree to which these CMC tools and electronic platforms can transfer the same face-to-face interpersonal communication, group learning, and group dynamics when learning and working together in an online setting. Social presence influences the way how the social interaction in OGL groups unfolds online, which, in turn, affects the learning outcomes (Tu and McIsaac, 2002; Zhao et al., 2014). Indeed, research has found social presence impacts group learning and group dynamics via social interaction (Tu, 2000) and vice versa; social interaction may reinforce social presence (Song and Yuan, 2015). Furthermore, Poth (2018) stated: “[d]etermining how to develop an individual’s ‘social presence’ within the learning environment is key to promoting a more engaging and supportive educational experience, in which students become more motivated and can attain more success” (p. 89) and Mykota (2017) appraised social presence as “the critical affective component and [...] one of the more important constructs in determining the level of interaction and effectiveness of learning in an online environment” (p. 137).

However, despite its importance, a precise definition of social presence has been under debate as presently a plethora of different definitions of social presence and incompatible measures for assessing it exist (Lowenthal and Snelson, 2017). This resulted in a situation that brings much confusion because definitions of social presence may differ so much that they seem to stem from totally different frameworks or theories. The lack of a precise definition and incompatible measures prevents the development of a coherent research field regarding social presence, its determinants and consequences, and its defining role in online group learning. This explains why social presence researchers have found equivocal results. For example, Giesbers et al. (2014) found no difference in social presence experiences when text-based CMC was compared to web-based video conferencing, whereas this difference was expected (Sallnäs, 2005). They also did not find that web-based video conferencing led to improved student performance and higher student learning experiences; in fact, student performance was worse, which contrasts with other research findings (e.g., Satar, 2013).

In this paper, we present our solution to resolve this undesired and complex situation. To that end, we went back to the original social presence theory as devised by the communication researchers Short et al. (1976) to show that although they themselves had a clear idea of social presence—namely “realness” of other persons in the interaction—their definition is nonetheless ambiguous and not operationalizable. Further, their measurement of social presence is questionable for two reasons: (1) there was no construct validation of the measurement instrument, and (2) the measurement instrument did not assess the degree of perceived “realness” of other persons but rather persons’ attitude toward a communication medium.

**Fig. 1:**
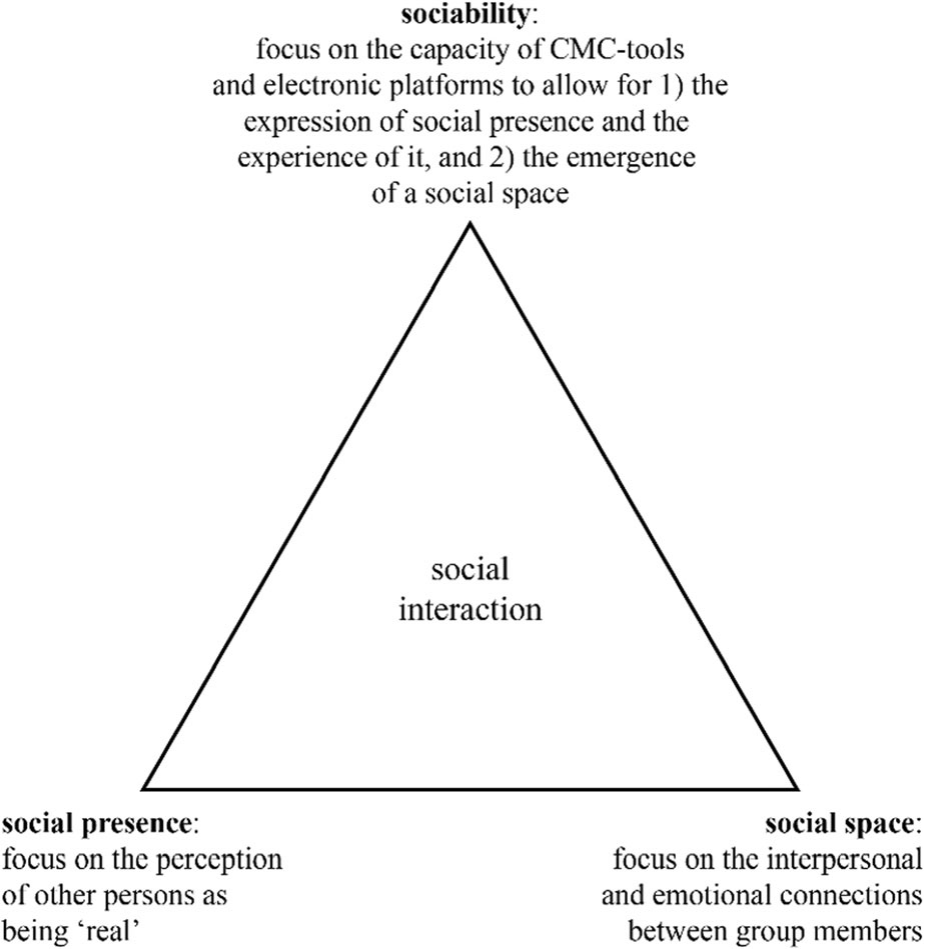
*The social presence, sociability, and social space triangle*

We, therefore, disentangled their social presence theory and (1) reformulated their social presence definition to enable an operationalization in line with their conceptualization of social presence; (2) departed from their technological determinism of social presence; (3) identified two other constructs, namely, sociability and social space. With respect to the first point, we reformulated social presence as the psychological phenomenon in which, to a certain extent, the other persons are perceived as physical “real” persons in technology-mediated communication enabled by CMC tools and electronic platforms (see also Kreijns et al., 2014; Weidlich and Bastiaens, 2017, 2019). With respect to the second point, Short et al. (1976) saw the physical attributes of the communication media completely determining the degree of social presence. As this technology determinism is nowadays much and rightly criticized (e.g., Gunawardena, 1995; Walther, 1993), we, therefore, propose “realness” of the other persons to be also determined by other factors as well. With respect to the third and last point, we define sociability as the capacity of CMC tools and electronic platforms to allow for the expression of social presence and the experience of it as well as for the emergence of a social space. As sociability is a capacity of CMC tools and electronic platforms, it is a medium attribute (Kreijns et al., 2002; Weidlich and Bastiaens, 2019). Social space is defined as the network of interpersonal relationships embedded in group structures of norms and values, rules and roles, and beliefs and ideals; a sound social space is manifested by sense of community, group climate, mutual trust, social identity, and group cohesion. These interpersonal relationships may exist in any group, for example, between the members of an OGL group or between participants in a learning network. Hence, social space is a group attribute (Kreijns et al., 2004). The three constructs, social presence, sociability, and social space, are interrelated with each other as they do not work in isolation: together, they influence how social interaction in groups is established and maintained. Figure 1 visually shows this as a triangle where the sides represent the interrelations and the vertices the constructs social presence, sociability, and social space; social interaction is positioned in the center of the triangle to reflect that it is affected by those three constructs. However, most social presence researchers—unwitting of the sociability and social space constructs—apply social presence theory wherein, in fact, the three constructs are collapsed into one “social presence” construct, causing a jingle-fallacy. This fallacy looms when two or more conceptually different constructs are erroneously assumed to be the same because they bear the same label. This jingle-fallacy explains the confounding situation.

This paper is outlined as follows: we start with the adoption of the original social presence theory by online distance educators to understand the interpersonal communication, group learning, and group dynamics in OGL groups and how this, in turn, affects learning outcomes (the “Social Presence and Online Group Learning” section). We continue by giving an overview of the many definitions and measures of social presence; the most frequently used social presence definitions and measures are elaborated. We point out that all these definitions and measures, however, created an undesirable confounding situation (“The Confounding Situation” section). We then noted that the confounding situation convinced some social presence researchers to “kill” social presence (the “How Further with the Confounding Situation?” section). Rather than “killing” social presence, we describe in the next section how we tried to resolve the confounding situation by disentangling the original social presence theory. We reformulate the definition of social presence and present two interrelated constructs of social presence: social space and sociability (the “Resolving the Confounding Situation” section). We end with a *Discussion*, a *Limitation*, and a *Conclusion* section.

## Social Presence and Online Group Learning

Short et al. (1976) devised social presence theory in order to understand interpersonal communication and relationship building in a business setting when using telecommunication media and how this affects the social influence communication partners may exert on each other. According to them, each telecommunication medium is characterized by the degree to which it can communicate verbal and nonverbal cues conveying socio-emotional information in such a way that the other persons involved in the communication are perceived as physical “real” and present. Accordingly, they defined social presence as the “degree of salience of the other person in the interaction and the consequent salience of the interpersonal relationships” (p. 65). Short et al. (1976) stated that social presence “varies between different media, it affects the nature of the interaction and it interacts with the purpose of the interaction to influence the medium chosen by the individual who wishes to communicate” (p. 65). They further stated that “Social Presence [is] a quality of the medium itself” (p. 65). Thereupon, they could categorize each telecommunication medium according to its degree of social presence by asking respondents to rate the communication media on four seven-point bipolar scales (Short et al., 1976, p. 66): (1) unsociable–sociable (2) insensitive–sensitive (3) cold–warm, and (4) impersonal–personal. Short et al. (1976) related two types of behaviors to social presence, namely, immediacy and intimacy behaviors. According to Short et al. (1976), immediacy is “a measure of the psychological distance which a communicator puts between himself and the object of his communication, his addressee or his communication” (p. 72). The concept of immediacy was developed by Wiener and Mehrabian (1968) in face-to-face settings; immediacy behaviors are the verbal and nonverbal approach behaviors that all simultaneously communicate warmth, availability, closeness, and interest. According to Short et al. (1976), intimacy behaviors are “a function of eye-contact, proximity, conversation topic and so on; changes in one will produce compensating changes in the others (…) eye-contact is generally sought after, but too much creates discomfort; for instance, eye-contact is reduced when people are placed very close together” (p. 53). Therefore, Argyle and Dean’s (1965) concept of intimacy—also developed in face-to-face settings—is an equilibrium theory postulating that communicators will reach an optimal level of “intimacy” in which conflicting approaches and avoidance forces are in equilibrium. Short et al. (1976) suggested that social presence of the communications media should be included in the list of factors contributing to intimacy.

Gunawardena (1995) was the first to see the potential of using social presence theory and its connection with intimacy and immediacy behaviors in an online distance educational setting to explain student satisfaction with group learning in a text-based computer conference system called GlobalEd. Although she initially defined social presence in line with Short et al.’s (1976) conceptualization of social presence as “the degree to which a person is perceived as a ‘real person’ in mediated communication” (p. 151), she conceptualized social presence much broader. In her view, social presence is not only a perception but also something that can be created or cultivated by participants. Indeed, Gunawardena (1995) purported that the degree of social presence largely depends on how learners in the GLobalED conference system were able to create social presence: “although CMC is described as a medium that is low in non-verbal cues and social context cues, participants in conferences create social presence by projecting their identities and building online communities” (p. 163). This perspective on social presence as an ability is reiterated in another study: “[r]esearch on social presence and CMC has indicated that despite the low social bandwidth of the medium, users of computer networks are able to project their identities, whether ‘real’ or ‘pseudo,’ feel the presence of others online, and create communities with commonly agreed-upon conventions and norms that bind them together in exploring issues of common interest” (Gunawardena and Zittle, 1997, p. 11). These quotes also show that Gunawardena (1995) linked social presence with community building and social cohesion among participants: GlobalEd despite being “a medium that is low in social context cues, it can be perceived as interactive, active, interesting, and stimulating by conference participants” (p. 147) due to “the kind of interactions that take place between the participants and the sense of community that is created during the conference” (p. 147). As a result, Gunawardena (1995, p. 162) asserted that social presence is largely determined by the user’s perception of the medium of CMC as being “social” and not so by the attributes of the communication medium. It is, therefore, that she—in contrast to Short et al. (1976)—asserted that that “immediacy enhances social presence” (p. 151). She measured social presence by extending the four bipolar scales of Short et al. (1976) with 13 other bipolar scales resulting in 17 bipolar scales aimed at soliciting “student reactions on a range of feeling towards the medium of CMC” (p. 150) and, hence, are referred to as the Students” Personal Reactions to CMC scale. However, Gunawardena and Zittle (1997) later maintained that these 17 bipolar scales actually addressed the intimacy aspect of social presence, whereas their newly developed social presence measure is addressing the immediacy aspect of social presence. This latter measure has 14 five-point Likert scale items and is referred to as the Social Presence Scale (SPRES) (see Table 2). To summarize, Gunawardena (1995) saw social presence as a perception of the “realness” of the other persons, as an ability to project one’s identity in mediated communication, and as community building and social cohesion.

Gunawardena’s (1995) perspective on social presence and her findings started a whole new line of research in online distance education wherein the role of social presence is studied in online group learning. In particular, that research is focusing on the relationship of social presence and certain outcomes including the learner satisfaction (Aragon, 2003; Dajani, 2015; Hostetter and Busch, 2006; Moallem, 2015; Richardson and Swan, 2003; So and Brush, 2008), social/group climate (Akyol and Garrison, 2008; Rourke and Anderson, 2002; Tu, 2002a), participation and online interaction (Cui et al., 2013; Danchak et al., 2001; Jorge, 2010; Polhemus et al., 2001; Zhao et al., 2014), perceived learning (Caspi and Blau, 2008; Maddrell, 2011; Richardson and Swan, 2003; Swan and Shih, 2005), learning outcomes (Russo and Benson, 2005; Hostetter, 2013; Madrell, 2011; Shin, 2003; Wei et al., 2012), motivation (Bai, 2003; Robb and Sutton, 2014; Tao, 2009), community building (Lin et al., 2017; Rourke et al., 2001; Rovai, 2002), and dropout (Bowers and Kumar, 2015; Robb and Sutton, 2014).

## The Confounding Situation

### Conceptualizations and Definitions of Social Presence

Unfortunately, what followed this adoption of Short et al.’s (1976) social presence theory in online group learning is an expansion of new interpretations of what social presence is and what it determines. In fact, many conceptualizations and definitions of social presence have come into existence as well as measures for assessing it (Biocca et al., 2001b; Cui, 2013; Kreijns et al., 2014; Lowenthal and Snelson, 2017; Weidlich and Bastiaens, 2017). Table 1 gives an overview of many of these definitions without being exhaustive.

**Table 1:** *Definitions of social presence*

Authors	Definition
Abdullah (2004)	“a sense that online users have of the communicators being ‘real’ interlocutors with personalities and physical presence […]. In other words, an interlocutor’s [social presence] is like the impression one would have of him or her if that interlocutor were physically present in the communication” (p. 3)
Arbaugh et al. (2008)	“the ability of participants to identify with the community (e.g., course of study), communicate purposefully in a trusting environment, and develop inter-personal relationships by way of projecting their individual personalities” (p.134)
Belderrain (2006)	“the degree to which individuals perceive intimacy, immediacy, and their particular role in a relationship” (p. 149)
Biocca et al. (2001a)	“the moment-by-moment awareness of the co-presence of another sentient being accompanied by a sense of engagement with the other (i.e., human, animate, or artificial being)”
Garrison (2009)	“the ability of participants to identify with the community (e.g., course or study), communicate purposefully in a trusting environment, and develop interpersonal relationships by way of projecting their individual personalities” (p. 352)
Garrison et al. (2000)	“the ability of participants in a community of inquiry to project themselves socially and emotionally, as ‘real’ people (i.e., their full personality), through the medium of communication being used” (p. 94)
Gunawardena (1995)	“the degree to which a person is perceived as a ‘real person’ in mediated communication” (p. 151). “[t]he ability to project one’s identity” (p. 163).
Gunawardena and Zittle (1997)	“the degree to which a person is perceived as ‘real’ in mediated communication” (p. 8)
Hassanein and Head (2007)	“where the medium gives the user a sense of human warmth and sociability” (p. 690)
Jung et al. (2002)	“interaction between learners and instructors that occurs when instructors adopt strategies to promote interpersonal encouragement and social integration” (p. 153)
Kang et al. (2007)	“perceived depth of relationships with other learners and the community during e-learning.” (p. 2)
Kim (2011)	“the specific awareness of relations among the members in a mediated communication environment and the degree of proximity and affiliation formed through it” (p. 766)
Kozan and Richardson (2014)	“the degree to which participants feel affectively connected to one another” (p. 69)
Moreno and Mayer (2004)	“[a] feeling of being with and interacting with another social being” (p. 166)
Picciano (2002)	“a student’s sense of being in and belonging in a course and the ability to interact with other students and an instructor” (p. 22)
Ogara et al. (2014)	“the degree along some definable continuum of unsociable—sociable, insensitive—sensitive, cold—warm, and impersonal—personal” (p. 455).
Remesal and Colomina (2013)	“the result of constructive and evolutionary discursive group interaction which promotes the creation of a community feeling, the maintenance of positive relational dynamics, and the enhancement of self- and collective efficacy in front of the learning task, in such a way that the learning process is supported” (p. 258)
Russo (2000)	the degree to which a person is perceived to be ‘real’ in a technology mediated environment
Short et al. (1976)	“degree of salience of the other person in the interaction and the consequent salience of the interpersonal relationship” (p. 65)
Sung and Mayer (2012)	“the subjective feeling of being connected and together with others during computer mediated communication” (p. 1739)
Swan and Shih (2005)	“the degree to which participants in computer-mediated communication feel affectively connected one to another” (p. 115)
Whiteside (2017)	“a critical literacy for cultivating emotions and relationships, which ‘serves an influential role in advancing and sustaining successful, meaningful learning experiences’” (p.133)
Sallnäs (2005)	“the feeling that one is present with another person in a mediated environment.” (p. 438)
Shin (2013)	“a feeling of being in the company of someone and the perceptual illusion of nonmediation” (p. 941)
Tu (2002a)	“Social presence is the degree of person-to-person awareness, which occurs in the computer environment” (p. 34).
Tu and McIsaac (2002)	“[t]he degree of feeling, perception and reaction of being connected to other intellectual entities in online classrooms” (p.146)
Walther (1992)	“the degree to which users can feel others’ presence in the result of interpersonal interactions during the communication process” (p. 54).
Yen and Tu (2008)	“the degree of feeling, perception, and reaction of being connected by computer-mediated communication (CMC) to another intellectual entity through electronic media” (p.297)

There have been attempts to categorize the different conceptualizations and definitions of social presence. For example, Lowenthal (2010) ordered them on a continuum with on the one end definitions that emphasize perceptions of persons as being “real” and being “there” or “present” (these definitions overlap our definition of social presence, see the “Introduction” section) and on the other end definitions that emphasize the interpersonal connection between participants (these latter definitions overlap our definition of social space; see the Introduction section). According to Lowenthal (2010), most definitions are found in the middle of the continuum but retain some focus for both ends.

Still, some social presence definitions cannot be placed on Lowenthal’s social presence continuum because these definitions neither see social presence as a perception nor as an interpersonal connection. For example, Garrison et al. (2000)—building on Gunawardena’s (1995) perspective that social presence can be cultivated—reconceptualized it as an ability. Garrison (2009) even went one step further and saw social presence as progressing through the phases (1) acquiring a social identity, (2) have purposeful communication, and (3) building relationships.

Lowenthal’s (2010) social presence continuum also falls short regarding the social presence model (SPM) of Whiteside and Garrett Dickers (2016; see also Whiteside, 2015, 2017). They see social presence as a critical literacy for cultivating emotions and relationships, which “serves an influential role in advancing and sustaining successful, meaningful learning experiences” (Whiteside, 2017, p. 133). Their social presence model is used to organize the structure of an online or blended course and encompasses five components: (1) affective association (refers to the emotional connections among participants), (2) community cohesion (refers to the greetings, salutations, and the sharing of various resources), (3) instructor involvement (refers to the encouragement of students to participate in higher order thinking and critical inquiry and on developing relationships and social connections among students), (4) interaction intensity (refers to the level of interaction between students which becomes visible by quoting fellow students or paraphrasing their statements), and (5) knowledge and experience (refers to the prior knowledge and experiences students bring in the course community).

### The Measurement of Social Presence

The many different conceptualizations and definitions of social presence also contributed to the existence of many different measures for it. Table 2 gives an overview of the many social presence measures.

**Table 2:** *Measures of social presence*

Authors	Measure	Description	Sample items	Validation
Arbaugh et al. (2008)	*Name*: Community of Inquiry (CoI) survey *Assesses*: the CoI survey contains three dimensions; cognitive presence, social presence, and teaching presence. Only the social presence dimension is relevant	*Structure*: unidimensional *Number of items*: nine items *Answering categories*: five-point Likert scale *Note*: six of the nine items were derived from SPRES	1. Online or web-based communication is an excellent medium for social interaction 2. I felt comfortable participating in the course discussions 3. I felt comfortable disagreeing with other course participants while still remaining a sense of trust	*Validation*: PCA with oblique rotation (n=287) Cronbach α: 0.91
Biocca et al. (2001a)	*Name*: Networked Minds social presence questionnaire *Assesses*: mediated social presence	*Structure*: three-factor structure with sub-factors: 1. Co-presence (8 items): 1.1. Mutual awareness (6 items) 1.2. Isolation/aloneness (2 items) 2. Psychological involvement (20 items) 2.1. Mutual attention (8 items) 2.2. Mutual understanding (6 items) 2.3. Emotional contagion (6 items) 3. Behavior engagement (12 items) 3.1. Behavior interaction (6 items) 3.2. Mutual assistance (4 items) 3.3 Dependent action (2 items) *Number of items*: 38 items; the final scale did not encompass the sub-factor 1.2 *Answering categories*: seven-point Likert scales	1.1. Sub-factor: I was often aware of others in the environment 1.2. Sub-factor: I often felt as if I was all alone 2.1. Sub-factor: The other individual paid close attention to me 2.2. Sub-factor: My opinions were clear to the other 2.3. Sub-factor: When I was happy, the other was happy 3.1. Sub-factor: My actions were dependent on the other’s actions 3.2. Sub-factor: My partner did not help me very much 3.3. Sub-factor: The other could not act without me	*Validation*: Factor analysis and concurrent validity test (n=76) Cronbach α (1st factor): 0.74, n.r., respectively Cronbach α (2nd factor): 82, 0.87, 0.76, respectively Cronbach α (3rd factor): 0.75, 0.69, n.r., respectively n.r. = not reported
Gunawardena (1995)	*Name*: Students’ Personal Reactions to CMC *Assesses*: 1. Student reactions on a range of feeling towards the medium of CMC 2. Social climate among participants 3. Intimacy aspect of social presence	*Structure*: unidimensional *Number of items*: 17 bipolar scales *Answering categories*: five-point Likert scale *Note*: The scale extended Short et al.’s (1976) four bipolar items with an extra 13 items	1. Dull–stimulating 2. Impersonal–personal 3. Unsociable–sociable	*Validation*: none
Gunawardena and Zittle (1997)	*Name*: Social Presence Scale (SPRES) *Assesses*: immediacy aspect of social presence	*Structure*: unidimensional *Number of items*: 14 items *Answering categories*: five-point Likert scale	1. CMC is an excellent medium for social interaction 2. I felt comfortable interacting with other participants in the conference 3. I felt comfortable interacting with other participants in the conference	*Validation*: authors correlated the measure with six selected items of the Students’ Personal Reactions to CMC scale
Kang et al. (2007)	*Name*: Social Presence Scale *Assesses*: purports to measure online learners’ involvement	*Structure*: three-factor structure: 1. Co-presence (5 items) 2. Influence (7 items) 3. Cohesiveness (7 items). *Number of items*: 19 items *Answering categories*: five-point Likert scales	1st factor: The level of mutual interest seems high 2nd factor: We help each other solve difficult problems 3rd factor: My ideas help us proceed with group work	*Validation*: EFA with oblimin rotation (n = 305). Cronbach α: 0.74, 0.76, 0.73, respectively
Kreijns et al. (2020)	*Name*: Social Presence Measure *Assesses*: The “realness” of the communication partner	*Structure*: two-factor structure 1. Awareness of others (15 items) 2. Proximity with others (12 items) *Number of items*: 27 items *Answering categories*: five-point Likert scale *Note*: Conversion table is needed to convert total scores to Rasch person measures	1st factor: it feels like none of my fellow students are here 2nd factor: I strongly feel the presence of my fellow students	*Validation*: Rasch analysis (n = 82) Cronbach α: 0.92, 94, respectively
Kiliç Çakmak et al. (2014)	*Name*: Social Presence Scale *Assesses*: social presence in e-learning environments *Note*: The details of the Social Presence Scale were obtained by personal communication	*Structure*: three-factor structure 1. Interactive (7 items) 2. Cohesive (5 items) 3. Affective (5 items) *Number of items*: 17 items *Answering categories*: five-point Likert scale	1st factor: I address others with their names in the virtual environment 2nd factor: I share information with other people in the environment 3rd factor: I talk to my friends in the virtual environment about my personal issues	*Validation*: EFA with varimax rotation (n = 261); CFA (n = 200) Cronbach α: 0.76, 0.81, 0.75, respectively. Cronbach α of whole scale: 0.83
Kim (2011)	*Name*: Social Presence Scale *Assesses*: social presence in distance higher education	*Structure*: four-factor structure: 1. Affective connectedness (5 items) 2. Mutual attention and support (6 items) 3. Sense of community (4 items) 4. Open communication (4 items) *Number of items*: 19 items *Answering categories*: five-point Likert scale	1st factor: I was able to be personally close to other participants in the class 2nd factor: I tried to concentrate on our discussion 3rd factor: Even though we were not physically together in a traditional classroom, I still felt I was part of a group 4th factor: I enjoyed engaging in exchange of ideas with the other participants	*Validation*: EFA with direct oblimin rotation (n = 401), CFA (n = 497), and criterion validity (n = 221) Cronbach α: ranged from 0.82 to 0.87 Cronbach α of whole scale: 0.92
Lin (2004)	*Name:* Social Presence Questionnaire *Assesses:* social presence, social navigation, and awareness of others; (partly based on Picciano, 2002)	*Structure:* four-factor structure: 1. Perception of assistance (5 items) 2. Social comfort (4 items) 3. Social navigation (3 items) *Number of items:* 12 items *Answering categories:* five-point Likert scales	1st factor: I felt I came to know the other students in this past week online group activities 2nd factor: I was able to appreciate the humor of members of the group 3rd factor: Knowing what other members of the group did helped me know what to do	*Validation*: PCA with varimax rotation (n = 15) Cronbach α: 0.89, 0.92, 0.70, respectively
Moreno and Mayer (2002, 2004)	*Name:* Presence Questionnaire *Assesses:* sense of presence in virtual environments (based on Witmer & Singer, 1998)	*Structure*: unidimensional *Number of items:* 13 items *Answering categories:* seven-point scale	1. I felt that I was able to control events in the environment 2. My experience in the environment was consistent with my experiences in the real world 3. While I was in the environment, I experienced a strong sense of *presence*	Test-retest, item-to-total correlations, item-to-subscales correlations (Witmer and Singer, 1998). Cronbach α: 0.86
Picciano (2002)	*Name*: n.a. *Assesses*: social presence based on Inventory of Presence Questionnaire developed by the Presence Research Working Group (http://www.presence- research.org) and Tu (2002a)	*Structure*: unidimensional *Number of items:* 11items *Answering categories:* seven-point scale	1. I enjoyed the online course 2. I felt I got to learn a great deal about the other students in the online course 3. I did not find the online course threatening to me	*Validation*: none
Rourke and Anderson (2002)	*Name*: n.a. *Assesses*: social climate	*Structure*: unidimensional *Number of items*: six bipolar scale items *Answering categories*: four-point scale	1. Warm–cold 2. Untrusting–trusting 3. Impersonal–personal	*Validation*: pilot testing (n = 12)
Short et al. (1976)	*Name*: n.a. *Assesses*: social presence as “realness” of the other	*Structure*: unidimensional. *Number of items*: four bipolar scale items *Answering categories*: seven-point scale	1. Unsociable–sociable 2. Insensitive–sensitive 3. Cold–warm 4. Impersonal–personal	*Validation*: none
Sung and Mayer (2012)	*Name:* Online Social Presence Questionnaire (OSPQ)	*Structure*: five-factor structure: 1. Social respect (5 items) 2. Social sharing (5 items) 3. Open mind (3 items) 4. Social identity (4 items) 5. Intimacy (2 items) *Number of items*: 19 items *Answering categories*: five-point scale	1st factor: I feel a sense of presence when students or the instructor express their appreciations and gratitude about my question, idea, or opinion 2nd factor: When I study in online community, social relationship between instructor and I is important factor for successful learning 3rd factor: I feel a sense of presence when learners actively expose their emotions, opinions, or ideas 4th factor: I feel a sense of presence when students and teachers have a variety characteristic in my online community 5th factor: I feel a sense of presence when I share my personal life story with others in my online community	*Validation*: PCA with varimax rotation (n = 276), CFA (n = 276). Cronbach α: ranged from 0.85 to 0.86. Cronbach α of whole scale: 0.86
Wei, Chen, and Kinshuk (2012)	*Name:* n.a. *Assesses*: 1. Feelings of co-presence 2. Intimacy aspect of social presence 3. Immediacy aspect of social presence	*Structure*: three-factor structure: 1. Co-presence 2. Intimacy 3. Immediacy *Number of items*: 12 *Answering categories*: five-point Likert scale	1st factor: I felt like having others with me in the online classroom 2nd factor: I had a warm and comfortable relationship with others in the online classroom 3rd factor: I found myself respected by others in the online classroom	CFA (n=522). Cronbach α: 0.90, 0.87, 0.89, respectively.
Tu (1997)	*Name*: Chinese Students’ Personal Reactions to CMC	*Structure*: unidimensional. *Number of items*: 13 bipolar scale items *Answering categories*: five-point Likert scale *Note*: 11 items were re-used from SPRES (Gunawardena and Zittle, 1997) from which 3 items were from Short et al. (1976)	1. Stimulating–dull 2. Helpful–hindering 3. Unthreatening–threatening	*Validation*: none
Tu, 2002a:	*Name:* Social Presence and Privacy Questionnaire (SPPQ)	*Structure*: five-factor structure: 1. Social context (5 items) 2. Online communication (5 items) 3. Interactivity (4 items) 4. System privacy (7 items) 5. Feelings of privacy (6 items) *Number of items*: 27 *Answering categories*: five-point Likert scale	1st factor: Computer-mediated communication messages are impersonal (do not have human qualities or characteristics). 2nd factor: Computer-mediated communication messages convey feeling and emotion. 3rd factor: Using computer-mediated communication to communicate with others is pleasant. 4th factor: What is the likelihood that someone might read and/or re-post messages sent to or from you? 5th factor: How risky is it to share personal and sensitive topics online?	EFA with orthogonal and oblique rotation (n = 310) Cronbach α: 0.75, 0.85, 0.78, 0.84, 0.79, respectively. Note: The EFA removed 3 items from the pool of 30 items
Tu, 2002b	*Name:* CMC Questionnaire (CMCQ) This scale is the SPPQ of Tu (2002a)	*Structure*: five-factor structure: 1. Social context (4 items) 2. Online communication (5 items) 3. Interactivity (4 items) 4. System privacy (5 items) 5. Feelings of privacy (6 items) *Number of items*: 24 *Answering categories*: five-point Likert scale	1st factor: Computer-mediated communication messages are an informal and casual way to communicate 2nd factor: Computer-mediated communication messages convey feeling and emotion 3rd factor: Using computer-mediated communication to communicate with others is pleasant 4th factor: What is the likelihood that someone might read and/or re-post messages sent to or from you? 5th factor: How risky is it to share personal and sensitive topics online?	EFA with varimax rotation (n = 43) Cronbach α: 0.82, 0.88, 0.73, 0.76, 0.71 respectively. Note: The EFA removed 6 items from the pool of 30 items
Tu (1997)	*Name*: Chinese Students’ Personal Reactions to CMC	*Structure*: unidimensional. *Number of items*: 13 bipolar scale items *Answering categories*: five-point Likert scale *Note*: 11 items were re-used from SPRES (Gunawardena and Zittle, 1997) from which 3 items were from Short et al. (1976)	1. Stimulating–dull 2. Helpful–hindering 3. Unthreatening–threatening	*Validation*: none
Tu, 2002a:	*Name:* Social Presence and Privacy Questionnaire (SPPQ)	*Structure*: five-factor structure: 1. Social context (5 items) 2. Online communication (5 items) 3. Interactivity (4 items) 4. System privacy (7 items) 5. Feelings of privacy (6 items) *Number of items*: 27 *Answering categories*: five-point Likert scale	1st factor: Computer-mediated communication messages are impersonal (do not have human qualities or characteristics). 2nd factor: Computer-mediated communication messages convey feeling and emotion. 3rd factor: Using computer-mediated communication to communicate with others is pleasant. 4th factor: What is the likelihood that someone might read and/or re-post messages sent to or from you? 5th factor: How risky is it to share personal and sensitive topics online?	EFA with orthogonal and oblique rotation (n = 310) Cronbach α: 0.75, 0.85, 0.78, 0.84, 0.79, respectively. Note: The EFA removed 3 items from the pool of 30 items
Tu, 2002b	*Name:* CMC Questionnaire (CMCQ) This scale is the SPPQ of Tu (2002a)	*Structure*: five-factor structure: 1. Social context (4 items) 2. Online communication (5 items) 3. Interactivity (4 items) 4. System privacy (5 items) 5. Feelings of privacy (6 items) *Number of items*: 24 *Answering categories*: five-point Likert scale	1st factor: Computer-mediated communication messages are an informal and casual way to communicate 2nd factor: Computer-mediated communication messages convey feeling and emotion 3rd factor: Using computer-mediated communication to communicate with others is pleasant 4th factor: What is the likelihood that someone might read and/or re-post messages sent to or from you? 5th factor: How risky is it to share personal and sensitive topics online?	EFA with varimax rotation (n = 43) Cronbach α: 0.82, 0.88, 0.73, 0.76, 0.71 respectively. Note: The EFA removed 6 items from the pool of 30 items

Note: CMC = computer mediated communication; EFA = exploratory factor analysis; CFA = confirmatory factor analysis; PCA = principal component analysis

One of the social presence measures that have impacted much the development of other social presence measures is the Social Presence Scale (SPRES) of Gunawardena and Zittle (1997). It has inspired the very influential Community of (CoI) Inquiry model of Garrison et al. (2000), which is centered around three kinds of presences: in addition to social presence, cognitive and teaching presence are essential components of the CoI for developing a complete educational experience. Cognitive presence is the “extent to which learners are able to construct and confirm meaning through sustained reflection and discourse” (Garrison et al., 2001, p. 11), whereas teaching presence is “the design, facilitation, and direction of cognitive and social processes for the purpose of realizing personally meaningful and educationally worthwhile learning outcomes” (Anderson et al., 2001, p. 5). Arbaugh et al. (2008) developed a CoI survey instrument that assesses all three types of presences. Six of the nine items of the CoI survey instrument that form the social presence subscale were derived from SPRES. The CoI survey instrument is currently the most dominant measure in the CoI research community (e.g., Kozan and Richardson, 2014; Saadatmand et al., 2017; Traver et al., 2014). Nevertheless, this instrument has some psychometrical issues to the social presence subscale. While the Arbaugh et al. (2008) validation study revealed three dimensions for the social presence subscale in the CoI survey instrument (group cohesion, open communication, and affective expression; see also Díaz et al., 2010), an empirical study performed by Carlon et al. (2012) showed only two dimensions (social comfort and social experience). Kreijns et al. (2014) hypothesized that the social presence subscale probably consists out of three dimensions: social space, attitude, and social comfort; they, however, did not provide evidence for it. Recently, Abbitt and Boone (2021) performed Rasch analyses (Rasch, 1960; Bond and Fox, 2015) on the CoI survey instrument and found that the social presence subscale fitted the Rasch measurement model, suggesting a unidimensional scale. However, they did not report whether or not they performed a Rasch test to detect multidimensionality in the subscale to confirm the unidimensionality property.

What is made clear here is that the different measures for social presence make it hard or even impossible to compare the various findings of empirical research using one of these measures because they differ in the number of dimensions as well as what these dimensions are and because the measures do not necessarily assess “realness” of the other person but rather correlates of it (which were then referred to as dimensions). And even if the same dimension is considered (e.g., intimacy), items still may assess a different thing—do the four items of the intimacy dimension of the Wei et al. (2012) measure assess the same thing as the two items of the intimacy dimension of the Sung and Mayer (2012) measure? Again, here too, there is a potential jingle-fallacy.

## How Further with the Confounding Situation?

Kehrwald (2008) stressed that “[a] robust theory of social presence” is needed because it “benefits online teaching and learning by (1) advancing exploration of learning designs which utilize social processes, (2) promoting understanding of the social motivation of users, (3) improving the social affordances of telecommunications systems, and (4) enhancing research into social cognition, interpersonal communication, and theories of mind” (p. 89). But it has already been more than 13 years ago that Kehrwald (2008) remarked that “[d]espite the passage of 30 years since the genesis of social presence theory and more than 10 years since the identification of social presence as a key component of online learning [...], a single, shared understanding of social presence has not emerged” (p. 89). Given the latest developments regarding the social presence construct, we only can see a further increase in entropy of the understanding of what social presence is and how it is measured. Surely, “[t]he lack of a single, shared understanding of social presence is problematic in so far as the improvement of online teaching and learning may be hampered by unexplored assumptions about the nature, role, and function of this critical element of computer-mediated interaction” (Kehrwald, 2008, p. 89). In addition, Chen (2014) pointed out that “social presence [is] still elusive and difficult to define. Due to its ambiguity, many doubts exist related to the measurement of social presence. [...] future researchers should be cautious when advocating the importance of social presence in distance learning” (p. iii). Similarly, Kreijns et al. (2018; see also Biocca et al., 2003; Rettie, 2003) noted that “these issues [...] makes it difficult to compare current findings in the social presence domain and future research is at risk if the confounding situation continues to exist” (p. 32). It is in this context that Lowenthal and Snelson (2017) put forward the question of whether social presence is indeed influencing the degree of perceived learning and learning outcomes as stated by so many social presence researchers. In other words, how do we interpret the findings of the social presence research and their usefulness for designing online learning if different definitions and measures of social presence are used? It is this reason that social presence researchers tend to say to either “kill” social presence (Öztok and Kehrwald, 2017) or call for “future research on social presence and the development of effective instrumentation to measure the construct [that] should (a) have a solid conceptualization of social presence, (b) clearly report instrument validity and reliability in published research to allow for the use and selection of effective, valid, and reliable social presence instruments in the field, (c) ensure that media concerns and generalization are taken into consideration, and (d) assess social presence with diverse approaches” (Cui, 2013, p. 26-27). We have chosen to do the latter and outline in the next section our approach.

## Resolving the Confounding Situation

In order to get structure in the conceptual chaos surrounding social presence and its measurement, it seemed to us that the best way is to go back to the original theory of social presence as devised by Short et al. (1976) as to disentangle it. We found that their definition is ambiguous, not operationalizable, and their measurement of it questionable. As a result—and also previewed in the “Introduction” section—we (1) reformulated their social presence definition to enable an operationalization in line with their conceptualization of social presence; (2) departed from their technological determinism of social presence; and (3) identified two other constructs, namely, sociability and social space. In the next subsections, we will go more in-depth on these issues.

### Reformulating Short et al.’s Definition of Social Presence

To recapitulate, Short et al. (1976) defined social presence as the “degree of salience of the other person in the interaction and the consequent salience of the interpersonal relationship” (p. 65). It is easily seen that this definition actually comprises two parts, namely, “degree of salience of the other person in the interaction” and “[degree of] salience of the interpersonal relationship”; the second part is the consequent—but also the purpose—of the first part (Kreijns et al., 2014; Kreijns et al., 2018; see also: Vanden Abeele et al., 2007; Kehrwald, 2008). Because of the two-part definition, it is not clear to distance education researchers adopting social presence theory which part should be emphasized or is more important regarding the characterization of social presence. In addition, it is not clear what is meant by “salience,” which opens possibilities for multiple interpretations. Therefore, the definition of Short et al. (1976) is ambiguous and non-operationalizable as the meaning of the underlying latent construct of “salience” remains a point of discussion.

Notwithstanding their ambiguous definition, Short et al. (1976) themselves had a clear view of what social presence is. They emphasized the first part, which they saw as referring to the degree of physical “realness” of the other persons when communicating over communication media that is restricted in transferring verbal and non-verbal cues. This is evidenced by the fact that Short et al. (1976) did explicitly mention on several occasions in their book *The social psychology of telecommunications* that the degree of social presence is dependent on the degree to which the other person is perceived as a “real” person. For example, on page 73, they stated that for telephone, even when immediacy communication behaviors vary by an interlocutor, the “degree to which he is perceived as a ‘real person’—the Social Presence afforded by the telephone — will be the same.” On page 74, they pointed to a study of Champness (1973), who evaluated a commercial video system by asking respondents to judge this system by means of a questionnaire containing items like “It provides a great sense of realism” and (negative worded) “People on the other end do not seem ‘real’.” Lastly, on the same page, they clarified that “Social Presence depends upon not only the visual, non-verbal cues transmitted but also more subtle aspects such as the apparent distance of the other (influenced, perhaps, by voice volume) and the ‘realness’ of the other (influenced, perhaps, by the fidelity of speech reproduction).” The strongest evidence is when Short et al. (1976) expressed their expectation that “[i]t is within the scope of foreseeable technology to reconstitute by electronic means a virtual three-dimensional representation of an individual who is hundreds of miles distant” (in the *Preface* of their book, p. v). They, apparently, saw such three-dimensional representation as the highest form of fidelity of physical realness of the other. Nowadays, 3D holographic representation of a person has already become a reality (ARHT Media, 2020).

Some social presence researchers do agree with this “realness” aspect of the person and have formulated similar definitions. In addition to the earlier mentioned researchers (see Table 1), we mention Jacobson (2001), who defined social presence as the “[s]ense of being perceived as real when participating in a computer-mediated environment” (p. 653) and Kear (2010), who related social presence “to the need for users to feel connected with each other and to perceive each other as real people” (p. 541). She saw social presence both as perceiving each other as “real” and the need to feel connected.

Regarding the non-operationalization of the definition, if then Short et al. (1976) saw social presence as “realness” of the other persons in the communication, a definition that would incorporate this would make the it operationalizable, as “realness” of the other persons would be the underlying latent construct to be assessed. Therefore, we reformulated the Short et al. (1976) definition as the psychological phenomenon in which, to a certain extent, the others are perceived as physical “real” persons in technology-mediated communication enabled by CMC tools and electronic platforms (see also Kreijns et al., 2014; Kreijns et al., 2018; Weidlich and Bastiaens, 2017, 2019). It, thus, is a sense of other persons being “present” in the “here” and “now” even if this is not true. In other words, social presence is the illusion of being together with other persons as if the communication is not mediated at all by communication media (cf. Lombart and Ditton, 1997).

### Short et al. Measurement of Social Presence

As indicated above, Short et al. (1976, p. 66) used four seven-point bipolar scales to categorize each telecommunication medium according to its degree of social presence. They based their scale on general research on attitudes that is usually assessed by means of a series of bipolar scales (Osgood et al., 1957). The four bipolar scales were: (1) unsociable–sociable, (2) insensitive–sensitive, (3) cold–warm, and (4) impersonal–personal. The more sociable, sensitive, warm, and personal a communication medium is perceived, the higher the social presence experienced. They concluded that face-to-face meetings have the highest degree of social presence, then closed-circuit video channels followed by audio channels, with telephone having the lowest degree of social presence.

However, Short et al. (1976) social presence measure has some psychometrical flaws. First, they did not construct-validate the four bipolar scales and have taken for granted that these scales measure the degree of social presence of the communication medium. Such a position raises the question whether the bipolar scales really are measuring social presence and not something else. Second, the lack of construct validation also means that it is unsure whether the full breadth and depth of social presence are captured by these four bipolar scales (Tu, 2002a; see also Messick, 1996). Third, Short et al. (1976) maintained that social presence is an objective quality of the communication medium. Yet, the four bipolar scales measure the subjective quality of this medium because attitudes are assessed toward the communication medium, which would then be “social presence.” According to Bradner and Mark (2001), the four bipolar scales form a relative measure of social presence and not an absolute one which most researchers actually need (p. 158).

In conclusion: the four bipolar scales of Short et al. (1976) to measure social presence is questionable for two reasons: (1) there was no construct validation of the measurement instrument, and (2) the measurement instrument did not assess the degree of perceived “realness” of other persons but rather persons’ attitude toward a communication medium. Kreijns et al. (2020), therefore, proposed a measure of social presence that operationalizes “realness” of the other persons in the communication in accordance with the reformulated social presence definition. Rasch analyses (Rasch, 1960; Bond and Fox, 2015; Wright and Masters, 1982) were performed to establish construct validity. Thereby, as Short et al. (1976) pointed out that they “conceive of Social Presence as a single dimension representing a cognitive synthesis of all the factors […] as they are perceived by the individual to be present in the medium” (p. 65), Kreijns et al. (2020) were aiming at a unidimensional social presence measure. However, the Rasch test for multidimensionality revealed two dimensions for social presence, namely, “awareness of others” and “proximity with others”; the former dimension indicates low perceptions and the latter higher perceptions of social presence. The dimensions match some of the social presence definitions in the literature. Definitions supporting one or both dimensions were given by Biocca (1997), who saw awareness of the others as “the minimum level of social presence [which] occurs when users feel that a form, behavior, or sensory experience indicates the presence of another intelligence,” Kim (2011) who defined it as “the specific awareness of relations among the members in a mediated communication environment and the degree of proximity and affiliation formed through it” (p. 766), and McLeod et al. (1997) defined social presence as “The degree of tangibility and proximity of other people that one perceives in a communication situation” (p. 708).

### Determinants of Social Presence

Short et al. (1976) devised their social presence theory in the late 1970s. It is, therefore, important to be aware that at that time there was no Internet yet, and all communication happened through communication media that involved the transfer of either video, audio, or a combination of both. In addition, the communication was unbuffered, meaning that it could not be stored and, thus, only immediate (synchronous or real-time) communication was possible. Hence, social presence could only be experienced during these immediate communication episodes while using these media. Therefore, it is—because of this setting—not surprising that Short et al. (1976) were inclined to state that the objective qualities of the communication medium are determining the degree of social presence.

However, as technology became more advanced and the advent of the Internet in the beginning in the early 1980s, other forms of communication media emerged that run on computers. Hence, this type of communication through computers was referred to as computer-mediated communication (CMC). The benefit of CMC is that it can be buffered and stored, meaning that it makes delayed (asynchronous or time-deferred) communication possible. CMC is also mainly text-based, as the usual CMC tools are email, online forums, and instant messaging. Delayed communication allows online distance learning to happen independently of time and place (Chandrasekaran et al., 2016; Harasim, 2012); the same applies to online group learning (Resta and Laferriere, 2007). In this situation, Gunawardena (1995; see also Gunawardena and Zittle, 1997) studied social presence experiences in the GlobalEd computer conference system and found other factors were influencing social presence experiences of distance students (see also, Tu, 2002a, 2002b). Indeed, Walther (1993, 1996) already suggested earlier that other factors, that is, factors related to social context, demographics, the individual, the subject of the interaction, and the images that participants have from each other are, are more critical to social presence experiences. Thus, these researchers suggested that not the medium attributes but social factors determine the degree of social presence.

The two lines of research caused what Spears et al. (2000) called the “technological versus social determinism” controversy when they studied social-psychological effects of information and communication technology (ICT) and CMC from different theoretical perspectives that also included social presence theory. They found that, in general, these “theories tend to assume that ICTs’ effects are due to characteristics of the technology, or that these are constructed by social factors” (p. 8). Spears et al. (2000), however, concluded from their studies that “the diversity of social effects precludes that technology is singularly good or bad, and that technology determines the social effects. Conversely, social determinism cannot account for invariable technological effects: not every use of ICTs is as flexible as these theories claim. Moreover, social determinism often is relativistic, which restricts its power of prediction and practical use” (p.8). The two lines of research were also indicated by Kehrwald (2008), who named them the “media richness view” and the “relational view,” respectively (p. 91).

Today we see that technology has evolved even further and that it goes beyond mere text-based CMC. For instance, chat programs like WhatsApp allow for the exchange of semi-synchronous messages that, in addition to text, also may incorporate short footages, emoticons, and animation, which give users endless possibilities to manipulate how other users may perceive their social presence. The same is true for those programs that bear the umbrella term of social software (e.g., Facebook^1^, Instagram^2^, TikTok^3^). Also, due to the recent COVID-19 pandemic, we see a growth in the use of real-time video communication applications including Zoom^4^ and Teams meetings^5^. All these latter applications are not CMC tools in the strict sense but are more of an electronic platform wherein you can collaborate with other persons as well. Also, most of these applications may not run only on computers but also on mobile phones and other electronic devices. Not surprisingly, we see increasing use of them in online distance education and OGL.

All these technological developments and their usage in OGL, as well as the new insights on social factors that may influence perceptions of social presence in technology-mediated communication, necessitates that we have to depart from a solely technological determinism of social presence perspective as put forward by Short et al. (1976). We suggest that social presence is co-determined, on the one hand, by the physical attributes of the CMC tools and electronic platforms and, on the other hand, by a contingency of individual and social factors such as personality, motivations, social context, social processes, and so forth. In other words, we adhere to the perspective that social presence is only partly determined by technology attributes.

Social determinism may be emphasized when dealing with present-day electronic OGL-platforms like BrightSpace and Canvas where the communication is predominantly delayed and text-based. As a matter of fact, the dominant and preferred way of delayed communication is still text-based (Ansari and Phillips, 2011). Technological determinism may be emphasized when dealing with electronic group learning environments based on VR applications (e.g., Oksanen and Hämäläinen, 2013; see also Oh et al., 2018). In these environments, the human body is represented by avatars that may or may not be ultra-realistic. Ultra-realistic avatars are currently implemented in Facebook Reality Labs (FLR, 2021) to see what it means for social presence experiences. FRL stated that their “goal is to make virtual interactions feel as natural as in-person interactions. We call this ‘social presence.’ It’s the 3D-enabled feeling that you’re physically sharing the same space with someone else, even though you may be miles apart — and that you can communicate your ideas and emotions seamlessly and effortlessly. To accomplish that in VR, you need lifelike avatars — virtual stand-ins that faithfully reproduce your facial expressions, gestures, and your voice.” We believe that such ultra-realistic avatars would be entirely in line with what Short et al. (1976) projected of what social presence is.

### Two Related Constructs of Social Presence: Social Space and Sociability

From the above (and hinted in the “Introduction” section), we already made clear that social presence is interrelated with two other constructs implied by Short et al.’s (1976) social presence theory, namely, social space and sociability. In the following sections, we describe more in-depth what these constructs are.

#### Social Space

As pointed out, Short et al.’s (1976) definition can be seen as consisting out of two parts, namely: “degree of salience of the other person in the interaction” (the first part) and “[degree of] salience of the interpersonal relationship” (the second part). Thereby, the second part is the consequence of the first part. Short et al. (1976) saw the development of interpersonal relationships separated from the salience of the other person when they discussed the significance of knowing someone already when telecommunication media are used (Chapter 8). Thus, rather than being part of the same social presence construct as implied by the original definition, we consider this second part as a related but different construct than social presence. This related construct is designated to be social space (Kreijns et al., 2004), which we define as the network of interpersonal relationships embedded in group structures of norms and values, rules and roles, and beliefs and ideals. A sound social space is manifested by a sense of community, group climate, mutual trust, social identity, and group cohesion. As such, social space can be seen as a group attribute. This definition will now be elaborated.

In accordance with the social network perspective (Katz et al., 2004), we see each interpersonal relationship as a tie that connects two communicating persons whereby these ties may be weak or strong. Consequently, all interpersonal relationships span a kind of space; hence, we speak of a social *space*. As mentioned above, all the interpersonal/social relationships are embedded within the group’s norms and values, rules and roles, and beliefs and ideals (see also: Blanchard and Markus, 2004; Brook and Oliver, 2002). Thus, apart from the spatial structure, social space also has a cultural structure because norms and values, rules and roles, and beliefs and ideals are cultural artifacts developed and maintained by the group members. Forsyth (2013), taking up a different perspective, identified four structures, a normative structure, an affective structure, a communication structure, and a role structure, but essentially, these structures come down to the same cultural artifacts mentioned above. Within these structures, communicating persons may experience a shared social identity, group cohesiveness, connectedness with others, mutual trust, a sense of belonging, a sense of community, a social climate, and an open atmosphere. Hence, we speak of a *social* space.

If these qualities exist in a positive way, we may designate the social space to be sound, such that it becomes a space for learning, thereby enabling productive social interaction for collaborative learning. Indeed, Johnson and Johnson (2009, see also Haythornthwaite, 2002, Moore & Kearsley, 1996, Palloff and Pratt, 1999, 2005) pointed out that good interpersonal relationships and a sense of community need to exist in order for social interaction for collaborative learning to take place, and vice versa, the collaboration will reinforce the sense of community. With regard to mutual trust, Johnson and Johnson (1989) pointed to its importance: “[t]o disclose one’s reasoning and information, one must trust the other individuals involved in the situation to listen with respect” (p. 72). Moreover, mutual trust is needed because group members will not participate collaboratively if they do not know with whom they are communicating (Smith and Kollock, 1998). Therefore, “trust is a central element in the provision of both a safe environment for learners and the conditions for communication and collaboration” (Jones et al., 2006, p. 50). Hara et al. (2000) concluded that in OGL, strategies promoting the sense of connectedness and belonging are critical for the learner. In fact, “[s]ocial presence helps to realise collaboration through establishing a warm and collegial learning community to encourage participation and interaction” (Zhao et al., 2014, p. 807). In addition, “[a] good atmosphere of learning interaction will be created when learners can perceive a high degree of social presence” (Wei et al., 2012, p. 533).

However, all the qualities mentioned here are not independent of each other but for the greater part overlap each other. In particular, sense of community seems to have the biggest overlap with most of the mentioned qualities. Sense of community originates from the work of Sarason (1974), who defined sense of community as “the sense that one [is] part of a readily available, mutually supportive network of relationships upon which one could depend and as a result of which one [does] not experience sustained feelings of loneliness” (p. 1). Trust seems to be an element of group climate (Brahm and Kunze, 2012). Given all these qualities that reinforce the social interaction in OGL groups, it is not surprising that many social presence researchers see these qualities as “social presence”—see, for example, Gunawardena (1995) and Rourke and Anderson (2002).

#### Sociability

We already addressed the fact that Short et al. (1976) apparently saw social presence to be determined solely by the physical attributes of the communication media, thus, their capacity to transfer verbal and nonverbal cues that allow for, to a certain degree, social presence experiences. Their four bipolar scales were meant to measure this capacity. However, as we pointed out above, there was no construct validation performed on the scales. It, therefore, is the question whether the bipolar scales really measure social presence as “realness” of the other persons. We believe that these bipolar scales are referring to yet another construct which is designated to be sociability (Kreijns et al., 2002). We define sociability as the capacity of CMC tools and electronic platforms to allow for the expression of social presence, the experience of it, as well as for the emergence of a social space. As such, sociability is a medium attribute. The definition aligns with Walther’s (1993) social information processing theory that distinguishes between impression management and impression formation in CMC; the impression is the mental model one has over the other person. Through impression management, persons may give expression to their social presence, and by impression formation, persons may experience the social presence of other persons.

We have labeled this capacity sociability after one of the bipolar scales of Short et al. (1976), namely, the “unsociable–sociable”-bipolar scale. In the case of OGL, the CMC tools and electronic platform may be the electronic learning platform used by the OGL groups. These platforms include proprietary platforms such as Canvas^6^ and Brightspace^7^ or open-source platforms such as Moodle^8^ and Elgg^9^.

Sociability as a capacity of CMC tools and electronic platforms is considered an attribute of these and, therefore, can be designed. Kreijns et al. (2002) have designated the particular functionalities that make up the sociability of CMC tools and electronic platforms to be social affordances (see also Kirschner and Kreijns, 2005). A canonical example of a physical social affordance device is the coffee machine where people meet by chance and have some small talk, usually about daily things that happened outside office hours. Although small talk may not necessarily contribute to formal communication or to learning, they do have a function with respect to the development of trustworthy interpersonal relationships and therewith to the social space, which may be beneficial when a work-related conversation is needed or when group work has to be done. It is this kind of social affordances that we seek to be present in electronic OGL platforms (see also Weidlich and Bastiaens, 2019).

It is purported that social media (Facebook, Instagram, TikTok) is high on sociability because they possess many types of social affordances; hence, they are deemed “social media.” The kind of social affordances found in these social media encompass, among others, possibilities for self-expression, personalizing, the upload of videos and photos for sharing, commenting on messages of other persons, blogging, grouping, and the use of a variety of emoticons when instant messaging. However, while indeed the availability of these affordances explains why social media has become so popular (Gao et al., 2010), it is also the hedonic affordances that contributed much to their popularity (Li, 2011).

Hedonic affordances determine the hedonicity of an electronic platform which is yet another medium attribute; and in the case of an electronic OGL platform, a third medium attribute is educability (Kreijns and Kirschner, 2018). Hedonicity expresses the extent to which CMC tools and electronic platforms support pleasure and enjoyment experiences during the course of working on group tasks. Past research on the adoption of technological devices and software systems has shown that hedonicity is an important factor in the adoption of these devices and systems and their continuous usage (Davis et al., 1992; Nysveen et al., 2005). Educability is the capacity of the electronic OGL platform to facilitate group learning by providing the educational affordances to support it. Educational affordances encompass shared work spaces (e.g., a shared whiteboard, a shared text processor), collaboration tools (e.g., a mind map tool, an argumentation space), and tools for feedback and reflection. It is expected that electronic OGL platforms do possess a certain minimum of educational affordances; otherwise, these platforms will be rendered useless for group learning.

## Discussion

Social presence is an evocative and popular notion. There have now been decades of research into the concept and its many applications in different domains. Yet, this has not produced a clear understanding of the concept and caused a situation that brings much confusion preventing the development of a coherent research field regarding social presence and its defining role in OGL. Subsequently, there have been numerous calls for more rigor in social presence research (Biocca et al., 2003; Öztok and Kehrwald, 2017; Lowenthal and Snelson, 2017).

The purpose of this paper was to disentangle social presence theory as devised by Short et al. (1976). We found, in essence, a strikingly severe case of the jingle-fallacy. This undesirable state of research, first formulated by Thorndike (1904), has been found to be present in many areas of psychology (e.g., Higgs and Lichtenstein, 2010; Larsen and Bong, 2016; Weidman et al., 2017) and appears to apply to social presence research as well. Indeed, by the disentanglement of social presence theory, we identified two other constructs, social space and sociability, that are closely linked with social presence yet separated from it. Nevertheless, social presence researchers often treat them as one, as if they all were “social presence.”

Potential consequences of this are, at best, an inconvenience for emerging scholars who are not steeped in the literature, at worst, effectively obstructing cumulative research of a concept that may be pivotal for understanding interpersonal effects in OGL contexts that are increasingly technologically mediated.

Although our work is not the first of its kind, the goal of this paper was to provide something the previous work has not, a possibility to integrate and incorporate the myriad of ways social presence has been defined in the past. For example, Lowenthal’s (2010) continuum was an elegant statement of a foundational problem in the literature on social presence. Due to its bipolar structure, however, it did not account for the one conception of social presence that has been so foundational in its history; the idea of intrinsic qualities of the medium or the environment to foster these interpersonal experiences. That is, it did not account for the prevalent conflation of social presence with sociability and social space.

Instead of merely showing what is out there and pointing to the deficiencies of the literature, we aimed to provide a way out, a framework through which we can better understand past research. Through this, we avoid the tendency of defiling or invalidating this large body of research and instead provide one step toward resolution. We found that definitions and measurement of social presence can be allocated in a space between three interrelated constructs, sociability, social presence, and social space. With this framework at hand, we can better understand the many correlates and effects that have been attributed to social presence but that may, in fact, be better attributed to sociability or social space.

As an example, let us consider the elusive connection of social presence to learning achievement. This connection is claimed by many (Wei et al., 2012; Molinillo et al., 2018), but convincing evidence is quite rare (Chen, 2014). Upon further reflection, one also notices the lack of a plausible mechanism for this connection. Why should the “realness” of the communication partner—or the illusion of non-mediation—exert causal influence on cognitive learning achievement? On the other hand, we have the notion of social space, which encapsulates group cohesion, mutual trust, and learning climate, aspects that have been consistently shown to be conducive to learning (Gunawardena, 1995; Rourke and Anderson, 2002; Rovai, 2002; Williams et al., 2006). A measurement of social presence that is entangled with social space will then naturally produce an association with learning achievement, which, however, should not be attributed to social presence. Confusions like these need to be avoided to make cumulative progress possible, both in our theoretical understanding of social presence and the practical application of this knowledge.

## Limitations

Although we aimed to provide a comprehensive review of the issues surrounding the definition and measurement of social presence, it remains possible that we have missed exemplary single studies or even lines of research that operate with coherent definitions and rigorous measures. However, we are confident that we have captured a large part of the most well-known and well-cited social presence research and, given the prevalence of issues in our reviewed sample, we find it unlikely that research we may have missed has fared significantly better in terms of defining and measuring social presence.

Importantly, our paper focused on the educational domain, more specifically, the realm of online distance learning, where OGL plays a large role. There are, of course, other areas of research that work with the concept of social presence, for example, VR, robotics, and conversational agents (see, e.g., Oh et al., 2018; Luria et al., 2019) and online shopping (Hassanein and Head, 2007; Ogonowski et al., 2014). In this sense, our literature review is limited. Yet, we have reasons to believe that these contexts are sufficiently different from most practices in online distance learning to warrant the exclusion of these research lines. Notably, they also grapple with the definition and measurement of their focal constructs (Slater, 2004; Hein et al., 2018).

Finally, our proposed solution of differentiating three central constructs appears sensible in light of the original definition of Short et al. (1976) and the research that has emerged since then. Still, it is of course possible that our rather narrow definition focusing on the “realness” of the communication partner will not be accepted by other scholars, who may prefer a richer conception of social presence, for example, one that encompasses affective and group climate-related aspects.

## Conclusion

Aiming for conceptual clarity, researchers concerned with social presence are encouraged to distinguish between the three major variables delineated in this paper. Thus, we recommend referring to sociability when we wish to talk about the capacity of CMC tools and electronic platforms when it comes to giving expression to one’s social presence and how one is perceiving or experiencing the other person’s social presence so to foster socio-emotional aspects of the learning experience. If, on the other hand, we consider the extent to which there is a network of interpersonal relationships in the OGL-group, we should refer to the term social space, which considers sense of community, group climate, mutual trust, social identity and group cohesion. Lastly, let’s only talk about social presence when we want to invoke the unique psychological phenomenon that we perceive other social persons as being physical “real” persons while using CMC tools and electronic platforms. Only then can we escape from the confounding situation surrounding social presence and build a coherent and cumulative research line.

## Footnotes

1 http://www.facebook.com

2 http://www.instagram.com

3 http://www.tiktok.com

4 http://www.zoom.us

5 https://www.microsoft.com/en-us/microsoft-teams/group-chat-software

6 https://www.instructure.com/en-gb

7 http://www.d2l.com

8 http://www.moodle.com

9 http://www.elgg.org
